# Can Hybrid Na–Air Batteries Outperform Nonaqueous Na–O_2_ Batteries?

**DOI:** 10.1002/advs.201902866

**Published:** 2020-01-19

**Authors:** Ziyauddin Khan, Mikhail Vagin, Xavier Crispin

**Affiliations:** ^1^ Laboratory of Organic Electronics Department of Science and Technology Linköping University SE60174 Norrköping Sweden

**Keywords:** aqueous, hybrid batteries, Na–air batteries, Na–O _2_ batteries, NASICON

## Abstract

In recent years, there has been an upsurge in the study of novel and alternative energy storage devices beyond lithium‐based systems due to the exponential increase in price of lithium. Sodium (Na) metal‐based batteries can be a possible alternative to lithium‐based batteries due to the similar electrochemical voltage of Na and Li together with the thousand times higher natural abundance of Na compared to Li. Though two different kinds of Na–O_2_ batteries have been studied specifically based on electrolytes until now, very recently, a hybrid Na–air cell has shown distinctive advantage over nonaqueous cell systems. Hybrid Na–air batteries provide a fundamental advantage due to the formation of highly soluble discharge product (sodium hydroxide) which leads to low overpotentials for charge and discharge processes, high electrical energy efficiency, and good cyclic stability. Herein, the current status and challenges associated with hybrid Na–air batteries are reported. Also, a brief description of nonaqueous Na–O_2_ batteries and its close competition with hybrid Na–air batteries are provided.

## Graphical abstract

Nonaqueous Na–O_2_ and hybrid Na–air are two different kinds of sodium metal‐based oxygen batteries. Herein, information about their charge storage mechanism and the fundamental advantage of hybrid Na–air batteries over nonaqueous Na–O_2_ batteries are provided. Further, the various essential components required and progress in the development of hybrid Na–air batteries are also discussed.

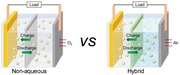

## 1 Introduction

**Figure 1:**
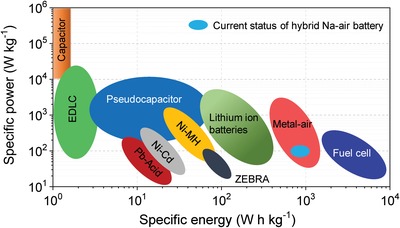
Comparative Ragone plot of various energy existing energy storage technologies and current status of hybrid Na–air battery.

Globally, energy and environmental issues are rife and prominent.^1^ Currently, the world is dealing with two great tasks: forthcoming reduction of hydrocarbon fuel resources aggravated by an increasing demand for energy due to the population and economic growths.[qv: 1c,2] In efforts to address those problems, the penetration of solar conversion technologies is the crucial target; which is today limited by the absence of a cheap battery technology. In that context, rechargeable batteries have been extensively studied for more efficient and environmentally benign energy‐storage systems.^3^ Among all myriad rechargeable battery systems, lithium‐ion batteries (LIBs) are considered the most promising technology for energy storage applications and also as a key system to build low‐carbon, sustainable electricity platform. LIBs have huge applications in all portable electronic gadgets, electric and hybrid electric vehicles and grid‐scale energy storage systems.^4^ But its large‐scale production for grid application is uncertain since there is a controversial debate about lithium availability and cost because battery industry requires almost 50% of the available resources of lithium.5f Additionally, limited Li‐ion conductivity in aprotic electrolytes and poor safety may also cause problems for its large‐scale utilization. Those drawbacks motivate researchers to find new energy storage technologies alternative to LIBs among which rechargeable metal–air batteries emerge as a new promising class of electrical storage (**Figure** **1**).

Typically, metal–air batteries (Li or Na) exhibit higher theoretical specific energy than LIBs which can make metal–air battery systems attractive and useful for hybrid and hybrid electric vehicles.^6^ Electrochemical power devices based on metal as anode and oxygen as a cathode active material have the highest energy density because the latter is not stored inside the device but is accessible from the environment. The theoretical specific energy of lithium–air batteries (LABs) is comparable with the theoretical specific energy of gasoline.[qv: 5c,7] The air cathode performance limits the cell capacity jeopardizing the commercial success of LAB technology. First, there is the consumption of solvent during the discharge process in the cathode reaction for both alkaline and acidic aqueous electrolytes. Second, there is the incompletion of discharge due to the blockage of pore orifices/openings.^8^ Therefore, one of the possible avenues toward LAB performance enhancement is the cathode material architecture,^9^ which can keep the active lithium ion and oxygen transport and can be filled with a substantial amount of products of oxygen reduction reaction (ORR) without the pores blockage. Dual pore materials, proposed in a field of gas diffusion electrodes (GDEs) for fuel cells are promising for energy capacity increase.^10^ Third, there is the air cathode degradation. Delivering most of the cell energy, the air cathode has the most of the cell voltage drop.^11^ The accumulation of LiO_2_ during discharge process yielding a mixed products as well as high voltages on charging caused the decomposition of solvent in parallel to lithium peroxide reduction.^12^ The oxygen solubility and rate of diffusion became crucial factors for battery energy capacity. The use of electrolytes with high oxygen solubility and high oxygen diffusivity yields in an enhancement of cathode capacity.^8^, ^13^

Among the group I and II elements, sodium metal‐based battery technology has emerged as a potential alternative to lithium‐based batteries owing to its similar electrochemical properties, high natural abundance (sixth most abundant element in earth crusts, **Table** **1**), and its standard reduction potential (−2.71 V vs SHE) is 230 mV above to lithium (−3.04 V vs SHE) which enables cell to produce large potential and thus store a lot of energy. Moreover, its comparatively high electronegativity in combination with low atomic weight can provide high specific capacity up to ≈1200 mAh g^−1^ which can offer economically viable solution compared to Li (3860 mAh g^−1^) in many applications.^14^ The combination of metallic sodium as one reactant for the battery with the inexhaustible source such as oxygen as the other reactant at the cathode is the basic concept of the so‐called Na–O_2_ battery. The natural abundance of both reactants is a prerequisite for the realization of large‐scale and high‐energy rechargeable batteries with many possible applications including the most challenging for grid‐based technologies.[qv: 6c,15] Although, Li–O_2_ batteries exhibit the highest specific energy, they encounter major issues related to the low electrical energy efficiency (due to large charge/discharge overpotentials >1 V), insoluble discharge products, and complex side reactions.^16^ Contrary to Li–O_2_ battery, Na–O_2_ batteries display a better electrical energy efficiency with pure carbon cathode and the discharge product, which is metal superoxide, is more stable when the anode is metallic sodium.[qv: 14a,16b,17] In further advancement, two different kinds of Na–O_2_ batteries were designed with the main difference lying in the types of electrolytes used, i.e., either nonaqueous electrolytes or the combination of an organic and aqueous electrolytes separated by a membrane in an hybrid architecture for the cell.

**Table 1:** Abundance of different metals and their standard reduction potential5f

Metals	Reduction potential [V]	Natural abundance [ppm]
Lithium	−3.04	18
Sodium	−2.71	22 700
Magnesium	−2.37	27 640
Calcium	−2.87	46 600
Aluminum	−1.66	83 000

Although significant advancements on both battery architectures have been achieved during the past few years, still major challenges remain unsolved. In this report, we review the recent progresses for the development and advancement in the area of sodium metal‐based air battery systems and attempt to summarize and answer critical questions. First, the concept and operation principle of sodium metal‐based air batteries that include nonaqueous Na–O_2_ battery and hybrid Na–air battery will be discussed. We will clarify the difference in their cell design and their electrochemical redox processes followed by a brief discussion on Na‐ion conducting membrane and anode. Second, the relevant published reports will be briefly explained which mainly involves different explored air electrodes for hybrid Na–air battery. Finally, a brief discussion will be provided whether hybrid Na–air cell can outperform the nonaqueous Na–O_2_ cell and the challenges associated with it.

## 2 Concept and Feature

### 2.1 Types of Cell and Its Design

**Figure 2:**
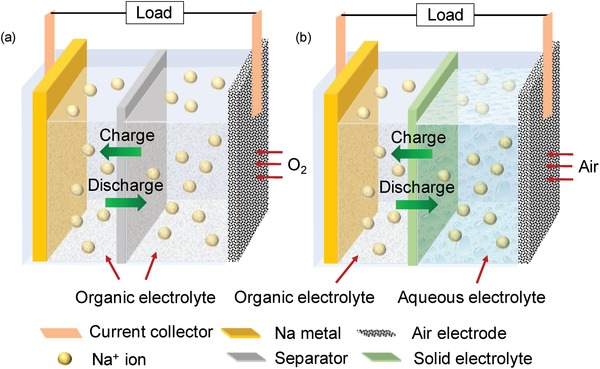
Schematic illustration of a) nonaqueous Na–O_2_ cell and b) hybrid Na–air cell.

In recent years, mainly two different types of sodium metal‐based air battery systems have been studied, namely, nonaqueous (often termed as Na–O_2_ battery) and hybrid batteries (often termed as Na–air battery). The use of O_2_ and air simply means the sources used for oxygen supply. In nonaqueous Na–O_2_ battery usually pure oxygen is supplied whereas in hybrid cells, air works as oxygen source. The cell structure of both types has a slightly different design and performance as illustrated in **Figure** **2** and **Table** **2**. A typical, nonaqueous Na–O_2_ battery is built up by the combination of metallic sodium as negative electrode and highly porous electrode as gas diffusion electrode (commonly known as electrocatalyst or air cathode).[qv: 6c] These two electrodes are separated by polymeric separator and immersed in an organic electrolyte (Figure 2a). On the other hand, the hybrid Na–air cell involves the utilization of two electrolytes: an organic electrolyte as anolyte and an aqueous electrolyte as catholyte. The demonstrated configuration includes a Na‐ion conducting solid electrolyte membrane which separates the negative compartment to positive compartment having different electrolytes (Figure 2b). The negative compartment design is the same as in conventional Na–O_2_ batteries whereas, the positive electrode is inserted in aqueous electrolyte. The solid electrolyte membrane is used to avoid the direct contact between the metallic Na and the aqueous electrolyte; which would lead to major energy loss through the spontaneous exothermic reaction. Hence, the solid electrolyte membrane only allows the transport of Na^+^ ions from the positive compartment to the negative and vice versa. Additionally, it also helps to prevent the growth of dendrites between the two electrodes which would lead to short circuits. In general, the energy storage mechanism in both cells involves the dissolution and plating of sodium metal at negative electrode during discharge and charge processes, respectively. However, on positive electrode ORR and oxygen evolution reaction (OER) takes place, when discharge and charge the cells.

**Table 2:** Comparison table between nonaqueous Na–O_2_ and hybrid Na–air battery

	Nonaqueous Na–O_2_ cell	Hybrid Na–air cell
Separator	Polymeric such as Celgard 3501	NASICON
Discharge product(s)	NaO_2_ and/or Na_2_O_2_	NaOH
Solubility of discharge product in electrolyte	Insoluble	Soluble
Total overpotential gap	High	Low
Electrical energy efficiency	≥80%	≤80%
Cyclic stability	Poor	Good
Safety	Satisfactory	Reasonable

### 2.2 Charge–Discharge Reactions

Generally, the energy storage mechanism in both types of cell involves the oxidation of metallic sodium into the sodium metal cation (Na^+^) and the release of an electron at the negative electrode during discharge. The electron moves to the counter electrode through the external circuit whereas the oxidized Na^+^ ion moves through the electrolyte to reach the counter electrode. The generated electron reduces the oxygen by ORR at the surface of the air electrode and the negative intermediate of the ORR is balanced by the Na^+^ ion to form a discharge product. On the contrary, upon charging, electroplating process of sodium occurs at negative electrode; while OER takes place at the air electrode. The discharge products are sodium peroxide (Na_2_O_2_) or sodium superoxide (NaO_2_) for the nonaqueous Na–O_2_ cell and sodium hydroxide (NaOH) for the hybrid Na–air cell. Nonetheless, the energy storage mechanism is clear in nonaqueous Na–O_2_ battery, but as far as the discharge products are concerned, their chemical nature is still a matter of debate.[qv: 6c,16b] The possible reactions pathways during discharge in nonaqueous Na–O_2_ battery can be explained as below:

At negative electrode

(1) $$\text{Na}\left(\text{s}\right)\to {\text{Na}}^{+}+{\text{e}}^{-}\;\left(E^{0}=+2.71\;\text{V}\right)$$

At air electrode, the possible all discharge products

(2) $${\text{Na}}^{+}+{\text{O}}_{2}+{\text{e}}^{-}\to {\text{NaO}}_{2}\;\left(E^{0}=-0.44\;\text{V}\right)$$

(3) $$2{\text{Na}}^{+}+{\text{O}}_{2}+2{\text{e}}^{-}\to {\text{Na}}_{2}{\text{O}}_{2}\;\left(E^{0}=-0.38\;\text{V}\right)$$

(4) $$2{\text{Na}}^{+}+1\text{/}2{\text{O}}_{2}+2{\text{e}}^{-}\to {\text{Na}}_{2}\text{O}\;\left(E^{0}=-0.76\;\text{V}\right)$$

Contrary to nonaqueous Na–O_2_ cells, sodium hydroxide is the only possible discharge product for a hybrid Na–air cell. Hence, the involved reaction steps can be explained using the following electrochemical equations (Equations (5) and (6))

At negative side

(5) $$\text{Na}\left(\text{s}\right)\to {\text{Na}}^{+}+{\text{e}}^{-}\;\left(E^{0}=+2.71\;\text{V}\right)$$

At positive side

(6) $${\text{O}}_{2}+2{\text{H}}_{2}\text{O}+4{\text{e}}^{-}\to 4{\text{OH}}^{-}\;\left(E^{0}=+0.40\;\text{V}\right)$$

### 2.3 Discharge Product in Nonaqueous Na–O_2_ Battery

**Figure 3:**
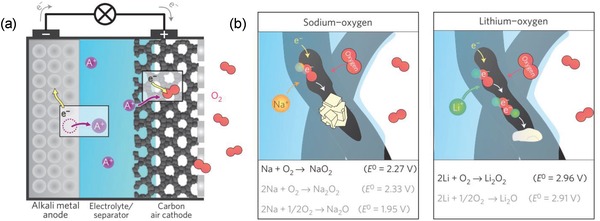
a) A rechargeable room‐temperature sodium superoxide (NaO_2_) battery. b) Different possible pathways for energy storage in nonaqueous Na–O_2_ and Li–O_2_ battery. In both metal–O_2_ battery formed discharge intermediate (O_2_^−^) reacts with Li^+^ ion and Na^+^ ion. In both cases, metal superoxide is formed but in case of Li, LiO_2_ is unstable and further reacts to form Li_2_O_2_ whereas, NaO_2_ is thermodynamically more stable. Other possible reactions are shown in gray color. Reproduced with permission.[qv: 6c] Copyright 2012, Nature Publishing Group.

A typical aprotic alkali metal–air battery (Li and Na based) and its working principle are illustrated in **Figure** **3**a.[qv: 6c] Indeed, Li and Na have quite close chemical reaction properties, however, their reactivity toward oxygen is enormously different.^18^ Li forms unstable lithium superoxide (LiO_2_) whereas, Na can form the stable NaO_2_ when reacting with oxygen during the discharge in a metal–O_2_ cell. Therefore, during discharge in a nonaqueous Na–O_2_ cell, there is a strong competition between the formation of NaO_2_ and Na_2_O_2_. Thermodynamically, the formation of Na_2_O_2_ is favorable (*E* ^0^ (Na_2_O_2_) = 2.33 V vs *E* ^0^ (NaO_2_) = 2.27 V), however, the synthetic path leading to NaO_2_ involves only one electron transfer per formula unit which makes it kinetically preferred in comparison to the two‐electron transfer toward Na_2_O_2_ formation (Figure 3b).[qv: 6c]

Certainly, there have been tremendous efforts to identify the discharge product in nonaqueous Na–O_2_ battery, but there has not been any conclusive evidence for any of the discharge product yet. Some reports suggest formation of NaO_2_ as a sole discharge product whereas, the Na_2_O_2_ was also found as discharge product in some studies.^19^ In a seminal study published by Bender et al., the authors estimated the free enthalpy of formation (Δ*G*) for Na_2_O_2_ (−449.7 kJ mol^−1^) and NaO_2_ (−437.5 kJ mol^−1^), and found that there is only −12.2 kJ mol^−1^ difference between the two discharge products. Since this difference is of the same order of magnitude as a hydrogen‐bond, it is extremely difficult to claim with certainty the thermodynamically preferred discharge product.[qv: 19d] It was also demonstrated that crystallite size also affects the formation of discharge product due to competition between surface and bulk energies.^15^ Kang et al. showed that Na_2_O_2_ is more stable in bulk phase; while NaO_2_ was found stable at nanoscale level where surface effects dominate. Moreover, the design of the cell also affects the formation of the discharge product as investigated by Bi et al. The authors observed NaO_2_‐based discharge product when the cell was built up with stainless‐steel chamber, however, Na_2_O_2_ was created when a glass chamber cell was used; while the rest of the conditions such as anode, cathode, and electrolyte remain the same.^20^ Since the thermal conductivity of glass (0.8 W m^−1^ K^−1^) is much lower than stainless‐steel (50 W m^−1^ K^−1^), the heat transfer from the reaction site to the chamber could be lead to various temperature distribution affecting the kinetics. But another origin could be that peroxide‐based discharge product usually forms when there is water in the system which might come from air or the decomposition of electrolyte. However, the authors concluded their case it might be due to the leakage in vacuum‐grease‐sealed glass chamber.^20^

**Figure 4:**
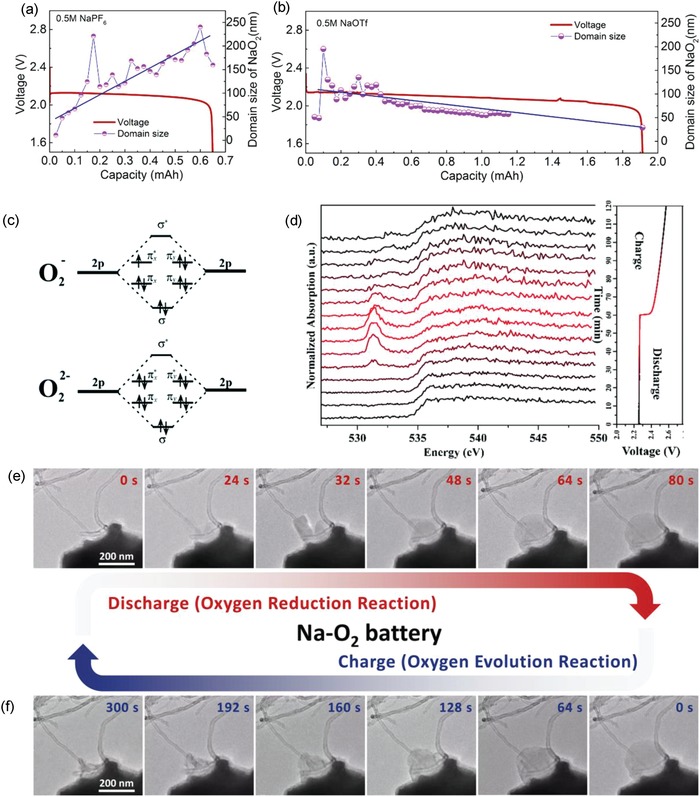
The estimation of average domain size of the formed discharged product (NaO_2_) for a) NaPF_6_ and b) NaOTf salt used in aprotic Na–O_2_ cell. Reproduced with permission.[qv: 21d] Copyright 2017, American Chemical Society. c) The electronic configuration of superoxide and peroxide anions. d) In situ XAS spectra of air electrode after cyclizing for 2 h in aprotic Na–O_2_ cell. Reproduced with permission.^23^ Copyright 2018, The Royal Society of Chemistry. In situ observation of e) formation and f) decomposition of discharge product using TEM in solid state Na–O_2_ nanobattery. Reproduced with permission.[qv: 21a] (https://doi.org/10.1021/acsenergylett.7b01273) Copyright 2018, American Chemical Society (further permissions related to the material excerpted to be directed to the ACS).

In nonaqueous Na–O_2_ battery systems, considerable efforts have also been given to understand and characterize the discharge product growth and chemical composition by various imaging and spectroscopic techniques.[qv: 6c,21] NaO_2_ on a discharged electrode was confirmed as product using Raman spectroscopy by Adelhelm's group.[qv: 6c] Typically, NaO_2_ exhibits a Raman band at 1156 cm^−1^, Na_2_O_2_ has two Raman bands in range of 700–825 cm^−1^ whereas, the Raman band at ≈225 cm^−1^ is ascribed to Na_2_O. Ex situ Raman spectra of discharged electrode showed clear bands corresponding to NaO_2_; which was also confirmed using ex situ X‐ray diffraction (XRD). Initially, ex situ techniques were utilized to recognize the discharge products and their morphology, however, those methods are not ideal for such sensitive discharge products that might get contaminated from the atmosphere. Therefore, to understand charge–discharge reaction mechanism of aprotic Na–O_2_ cell, in situ characterization techniques are highly desirable. Wang et al. have used CoO/CoP heterostructured nanosheets as bifunctional electrocatalyst to catalyze the charge–discharge reaction. Further, they utilized in situ XRD to recognize the discharge product. The authors found Na_2_O_2_·2H_2_O as the major product while NaOH and Na_2_CO_3_ diffractogram peaks were also observed in XRD.^22^ Liu et al. used in operando synchrotron radiation powder X‐ray diffraction (SR‐PXD) to probe the effect of electrolyte salt on the growth of crystalline NaO_2_ and quantify its formation.[qv: 21d] Na–O_2_ cells showed formation of NaO_2_ confirmed by in situ XRD however, note that the Na–O_2_ cell with NaOTf salt displayed higher discharge capacity than the cell built by using NaPF_6_ salt. Additionally, the average domain size of NaO_2_ was found smaller in cell having NaOTf salt (**Figure** **4**a,b) which helped in improving the discharge capacity. The authors proposed that the origin is due to a balance between nucleation and growth phenomena of crystallites during the discharge.[qv: 21d]

Soft X‐ray absorption spectroscopy (XAS), an effective tool to get information related to electronic and chemical structure of products by probing states near Fermi level, was also employed in operando by Xueliang's group.^23^ The two most common discharge products (NaO_2_ and Na_2_O_2_) in Na–O_2_ cell have different electronic configuration as presented in Figure 4c which helps to understand the formation and degradation of discharge product.^24^ O K‐edge XAS can be recorded by exciting O 1s electron to unoccupied orbital states of O 2p such as π* and σ* antibonding states.^25^ Peroxide species (O_2_^2−^) does not have π* transition because it has a completely filled π* orbital therefore, exhibits no associated XAS peak. However, superoxide species (O^2−^) has partially filled π* orbital which allows transition of electron to π* orbital and thus shows a corresponding transition peak at 532 eV (Figure 4d).^23^ XAS also shows a broad peak at 536 eV which can be ascribed to the transition of electrons to σ* antibonding state.[qv: 23,25a,26] The X‐ray absorption near edge structure (XANES) was also carried out by Landa‐Medrano et al. to understand the discharged state of Na–O_2_ cell and concluded that discharged product is composed from both NaO_2_ and Na_2_O_2_.[qv: 21e] Further, authors also confirmed the distribution of NaO_2_, Na_2_O_2_, and organic side products (OSPs) using soft X‐ray transmission microscopy (TXM) which also supported the XANES data. Analysis of spatial distribution of NaO_2_ and Na_2_O_2_ on cubical‐shaped discharged product showed uniform distribution of both NaO_2_ and Na_2_O_2_, however, OSP were distributed at the edges of cubes.

In addition to TXM, in situ transmission electron microscopy (TEM) and in situ Raman imaging techniques are also employed to study the charge–discharge mechanism in Na–O_2_ cells.[qv: 21a–c] Kwak et al. built a solid state Na–O_2_ nanobattery using Na metal as anode, carbon nanotube (CNT) as air electrode, and Na_2_O as solid electrolyte which was synthesized on the surface of metallic Na for in situ analysis of reaction mechanism.[qv: 21a] During discharge of cell (Figure 4e), formation and growth of NaO_2_ can be easily seen with time using time resolved in situ TEM. The nucleation of NaO_2_ starts as soon as the discharge process begins and after 48 s, the agglomeration of new formed nuclei occurs on the initial nucleation site. Interestingly, the decomposition and shrinkage of the discharge product was observed on charging the cell (Figure 4f). However, some traces of discharge product on electrode surface remained and are associated with the higher overpotentials during charging of the Na–O_2_ cells. Recently, Sun et al. studied extensively the charge mechanism of Na–O_2_ cell using Raman spectroscopy, energy dispersive X‐ray analysis, and XAS.[qv: 21f] Raman spectra of partially charged state of CNT‐PAN (polyacrylonitrile)‐based air electrode did not show any feature related to NaO_2_, however, two new Raman bands at 1080 and 1036 cm^−1^ were observed related to Na_2_CO_3_ and Na_2_O_2_·2H_2_O, respectively.^27^ Raman mapping suggested that Na_2_CO_3_ was mostly distributed at the outer surface of crystals whereas, Na_2_O_2_·2H_2_O was found at the inner surface of crystal which was also reported by Landa‐Medrano et al.[qv: 21e] XAS studies of discharged NCNT‐PAN‐based air electrode displayed three absorptions at 532, 532.9, and 534.4 eV ascribed to NaO_2_, Na_2_O_2_, and Na_2_CO_3_, respectively. Upon charging the same electrode, it was observed that the absorption peak corresponding to Na_2_O_2_ first increases (in partial charged electrode) and then reduced (on fully charged condition). On other hand, XAS peak related to Na_2_CO_3_ continuously grows and features related to NaO_2_ diminish with charging. It was also observed that major portion of Na_2_CO_3_ remained undecomposed even when the cell was charged up to 4.5 V. The carbonate‐based remnant, formed by reaction of sodium ions with electrolyte, can be possible origin of high overpotentials during charging of cell.

These various ex situ and in situ studies provide in‐depth insight about charge and discharge mechanism in nonaqueous Na–O_2_ cell. Although, NaO_2_ has been broadly accepted as major discharge product, yet, some authors also reported the concomitant formation of Na_2_O_2_ and Na_2_O_2_·2H_2_O and even Na_2_CO_3_, all those species are insoluble in organic electrolytes while during the charge some discharge products disappear, Na_2_CO_3_ on the contrary is stable even at high potential. This implies that a high energy is needed to decompose it; which results in high charge overpotentials, low round trip efficiency and poor rechargeability. The existing Na–O_2_ batteries also suffer with poor kinetics owing to the formation of microsized NaO_2_ cubes as discharged product. Taking the advantage of higher ionic and electronic conductivity of NaO_2_ than Na_2_O_2_, Khajehbashi et al. provide a unique solution to improve the kinetics and rate performance of cell.^28^ Since NaO_2_ is paramagnetic in nature, its combination with high spin electrocatalyst such as Co_3_O_4_ can lead the formation of NaO_2_ nanowires as discharge product. The formed 1D structure improves the electrical transfer in comparison to microsized NaO_2_ cubes or particles. This distinctive designed cell showed improvement in rate performance, high energy density, low charge overpotential, and good cyclic stability. Additionally, the use of organic electrolytes also has concerns for toxicity, flammability, economic, and environmental impact. Most of nonaqueous Na–O_2_ cell studies employed pure and dry O_2_ rather than air to prevent undesired parasitic reactions related to the components of ambient air such as water, CO_2_, CO, and N_2_. One possible strategy could be use of oxygen diffusion membrane that can selectively allow O_2_ from air while avoiding diffusion of the other components, as the case for aprotic Li–O_2_ batteries.[qv: 5c,29] However, inclusion of oxygen diffusion membrane in a cell will impede its high rate applications because water vapor can permeate through any membrane.^30^ Either way whether use of pure and dry O_2_ or incorporation of oxygen diffusion membrane will certainly increase the cost of the aprotic Na–O_2_ cell.

### 2.4 Discharge Product in Hybrid Na–Air Battery

To address the issues related with undecomposed discharge products, poor round trip efficiency and cost associated with organic electrolytes, mixed electrolyte systems have been proposed. The mixed electrolyte, commonly known as hybrid electrolyte, implies a separation of the electrolytes into two compartments in the cell with a solid electrolyte membrane blocking oxygen and water diffusion but transporting cations. The membrane separates the nonaqueous electrolyte as anolyte and an aqueous electrolyte as catholyte (cell design is already explained).^31^ The use of an aqueous electrolyte on the gas diffusion electrode enables the discharge product to be the soluble sodium hydroxide (4Na(s) + O_2_ + 2H_2_O → 4NaOH). The aqueous electrolyte also conveniently allows the source of oxygen to be air in contrast to the nonaqueous Na–O_2_ battery which requires supply of pure oxygen because of side products generated by water vapor in air.^31^ The energy density of an hybrid Na–air battery is not as high as hybrid Li–air battery (**Table** **3**) but there are various other advantages associated with this system. In addition to the low price of sodium and the use of cost‐effective material as current collectors,^32^ the essential advantage lies in the higher ionic conductivities of Na^+^ ions compared to Li^+^ ions in organic,^33^ aqueous, and solid electrolytes which is due to the higher mobility of Na^+^ ions in electrolyte solution.^34^ Since, Li^+^ ion has higher Lewis acidity than Na^+^ ions hence, there exist higher Coulombic interaction between Li^+^ cations and negatively charged species thereby leading to lower ionic mobility.^35^ Additionally, the solubility of sodium hydroxide in water is higher than lithium hydroxide which also helps improving the specific energy of the cell.[qv: 34b]

**Table 3:** Theoretical energy densities of hybrid Li–air and Na–air cells[qv: 34b]

Cell	Redox reaction	Cell potential [V]	Theoretical energy density [Wh kg^−1^]
Hybrid Li–air	Li + 1/2H_2_O + 1/4O_2_ → LiOH	3.45	3860
Hybrid Na–air	Na + 1/2H_2_O + 1/4O_2_ → NaOH	3.11	2090

Alike aprotic Na–O_2_ cell, NaOH is the only discharge product reported to date in hybrid Na–air cell, which is a soluble discharge product. Though, there does not exist any direct study in literature on discharge product of cell, post analysis scanning electron microscopy (SEM) of discharged electrode showed no sign of any residue related to discharge product as the case for aprotic Na–O_2_ cell.^36^ Also, TEM analysis of discharged electrode after 10 cycles did not show any trace of discharge product on electrode surface.^37^ However, ex situ XRD of discharge electrode after performing 1000 h charge–discharge displayed diffraction peak related to NaOH·H_2_O.^36^

## 3 Key Elements in Hybrid Na–Air Battery

**Figure 5:**
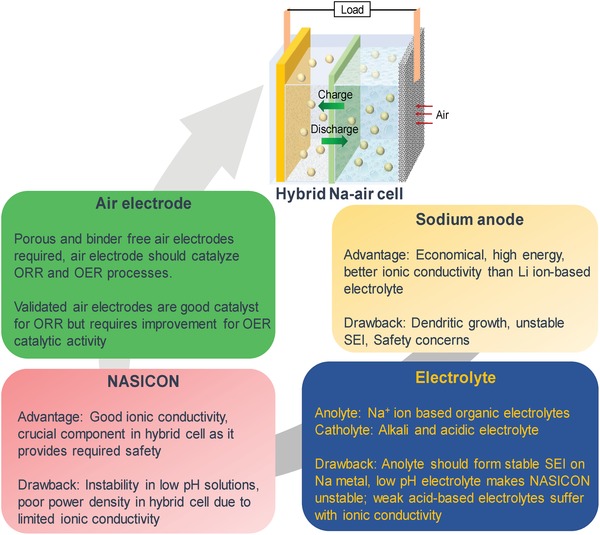
Scheme illustrating various essential components of hybrid Na–air cell with their advantages and disadvantages.

Like other metal–air batteries,[qv: 6a] hybrid Na–air battery has also four essential components namely anode, electrolyte (including organic and aqueous electrolyte), separator (solid electrolyte film called NASICON), and air electrode which is summarized in **Figure** **5**.

### 3.1 Solid Electrolyte Membrane as Separator

**Figure 6:**
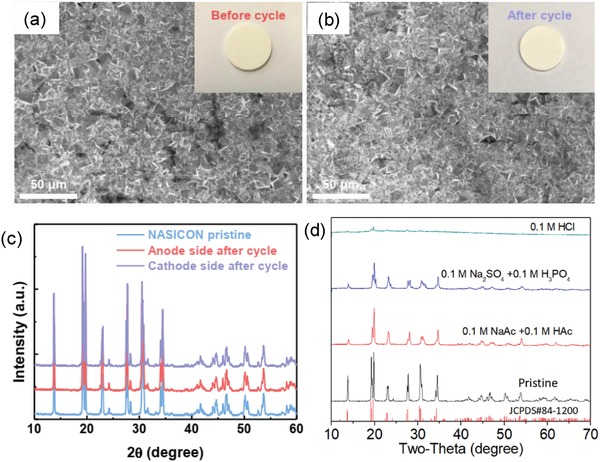
SEM images of NASICON film a) pristine; b) after being immersed in 5 m NaOH. c) X‐ray diffractogram pattern of pristine NASICON film and NASICON treated in 5 m NaOH. Authors examined the XRD at both side of the film. Reproduced with permission.[qv: 5e] Copyright 2019, The Royal Society of Chemistry. d) XRD of NASICON film in different acidic electrolyte and in pure acid. The diffractogram of NASICON film was completely changed when dipped in 0.1 m HCl solution which indicates the susceptibility of NASICON film in acidic solution. Reproduced with permission.^44^ Copyright 2018, American Chemical Society.

A solid electrolyte membrane separates the metallic Na compartment dipped in an organic electrolyte with the aqueous compartment to improve the safety in the hybrid Na–air cells. The solid electrolyte membrane should not only block the oxygen diffusion and promote the transport Na^+^ cations, as found in polyelectrolyte membranes (e.g., Nafion); but additionally, blocks even the diffusion of water from one to the other compartment (which is not the case in Nafion). Thus, it would be fair to say that the solid electrolyte membrane is a very crucial component of the cell. In most of the studies related to hybrid Na–air battery, NASICON was used as solid electrolyte membrane to separate aqueous and nonaqueous compartments. The abbreviation NASICON arises as NA from sodium (Na), S from super, I from ionic, and CON from conductor; which typically refers to a family of solid ceramics with general formula Na_1−_ *_x_*Zr_2_P_3−_ *_x_*Si*_x_*O_12_ (0 ≤ *x* ≤ 3).^38^ NASICON was first synthesized by Hong^39^ and Goodenough et al.[qv: 34g] in 1976. The transport of ions is caused by hopping of Na^+^ ions among interstitial sites of the NASICON crystal lattice.^38^ The resulting Na^+^ ion conductivity reaches on the order of 10^−3^ S cm^−1^; which is comparable to liquid electrolytes. NASICON can be synthesized by different synthetic methods such as solid state chemical synthetic route, sol–gel route, hydrothermal method, combustion method, and spark plasma sintering methods.^40^ Generally, NASICON crystallizes in rhombohedral structure for all compositions except (1.8 ≤ *x* ≤ 2.2) where it crystalizes in monoclinic polymorphs. The conductivity of NASICON is higher for monoclinic crystal lattice (*x* = 2). The performance of hybrid Na–air battery in terms of overpotential gap, power density, and cyclic stability will certainly depend on the stability and ionic conductivity of NASICON in alkaline and acidic medium. NASICON‐based materials are known to be stable in moist environment, however, they tend to form hydronium on the surface of NASICON by ion exchange when hot water is used.^41^ There is no direct study on the stability of NASICON in alkali electrolyte but after carefully inspecting the literature, NASICON appeared durable.^36^, ^42^ Liang and Hayashi synthesized NASICON with an ionic conductivity of 1.3 × 10^−3^ S cm^−1^ at room temperature and immersed the NASICON film in 1 m NaOH and organic electrolyte to investigate the change in its morphology.^42^ The ceramic film showed no change or deterioration in morphology in 1 m NaOH and 1 m NaClO_4_ in ethylene carbonate/dimethyl carbonate (EC/DMC) up to two days. However, the authors did not carry out any spectroscopic study to investigate the change in crystal phase or chemical bonding. Later, Khan et al. carried out NASICON's stability test by performing its XRD before and after galvanostatic charge–discharge cycles and found no changes in phase of NASICON.^36^, ^43^ Additionally, Park et al. investigated the stability test of NASICON in highly concentrated alkali electrolyte (5 m NaOH) solution while using it as a separator in rechargeable Na/Ni battery.[qv: 5e] Authors carried out 750 cycles charge–discharge of cell and then performed SEM and XRD to examine any changes in morphology and chemical composition of NASICON in 5 m NaOH. No significant change in morphology (**Figure** **6**a,b) and diffraction pattern (Figure 6c) was observed before and after 750 cycles. Moreover, Kang et al. showed the vulnerability of NASICON films when exposed to highly acidic solution (0.1 m HCl) (Figure 6d).^44^ At low pH solutions, oxide dissolution and ion exchange between Na^+^ and H^+^ occur and destabilize NASICON.^45^ NASICON is a good choice for separator as solid electrolyte in hybrid Na–air cells, yet it has limitations for charging cells at higher current densities due to its limited ionic conductivity. Also, an interfacial layer can be formed between the organic electrolyte and NASICON which enhances the overall resistance of the electrolyte and total internal resistance of cell. To understand the details of the process associated with NASICON requires more in‐depth investigations; for that purpose, operando spectroscopic methods will give powerful insight.

### 3.2 Anode

Like in other battery systems, the anode also plays an important role in optimizing the performance of the hybrid Na–air cell. Metallic Na is more chemically reactive and undergoes easier orientation growth than metallic Li and thus, the growth of Na dendrites are quite substantial during the cycle of plating and dissolution of Na associated with charge and discharge of the hybrid Na–air cell.^46^ These dendrites can reduce the performance of the cell and lead to serious safety concerns. The dendritic growth in hybrid Na–air cell can be overcome either by enabling the formation of stable solid electrolyte interphase (SEI) layer on the Na anode or by strategically designing the metallic Na anode. In most of the study reported so far metallic sodium has been used as anode.

**Figure 7:**
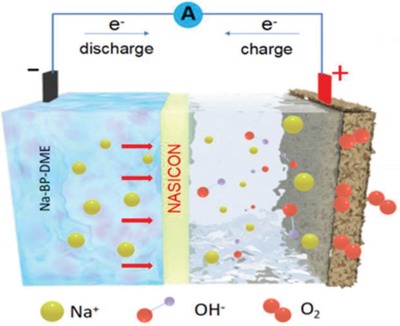
Schematic illustration of hybrid Na–air cell with liquid anode. Metallic sodium was dissolved in biphenyl and dimethoxyethane to form all liquid Na‐based anode. Reproduced with permission.^47^ Copyright 2018, Elsevier Ltd.

Liang et al. reported a modified approach and uses liquid anode to tackle the Na dendritic growth issue.^47^ The metallic sodium was dissolved in to mixed organic solvents (biphenyl and ethers) to provide an negatively charged biphenyl balanced by sodium cation with both electronic and ionic conductivities of the order of 10^−3^ S cm^−1^. This sodium‐based liquid anode was separated from the air electrode by the solid electrolyte NASICON as shown in **Figure** **7**. The cell exhibited lowest overpotential gap (0.14 V), high round trip efficiency (95.3%), 39 mW cm^−2^ of maximum power density, and exceptional cyclic stability. This outstanding performance of cell was attributed to the high ionic and decent electronic conductivity of the liquid anode, as well as the low interfacial resistance between the liquid anode and the solid electrolyte. Overall, the proposed system can be a potential alternate to avoid the usage of direct metal in metal‐ion or metal–air battery systems. The dissolution of the Na metal in contact with biphenyl produces a negative radical biphenyl stabilized with a sodium cation. The negatively charged biphenyl is then the reactant in the battery; thus the battery concept resembles the organic–air battery proposed recently by Cong et al.^48^

### 3.3 Electrolyte

Hybrid Na–air batteries are built up with two different electrolytes namely, organic and aqueous electrolytes. Sodium‐based salts such as NaCF_3_SO_3_, NaClO_4_, etc., are dissolved in organic solvents such as tetra ethylene glycol dimethyl ether (TEGDME) and EC/DMC and used as anolyte. The organic electrolytes are involved in the formation of SEI at anode which should allow deposition and stripping of Na^+^ ions. SEI forms mainly due to the decomposition of organic electrolytes at lower potential on anode and electrolyte interface.^49^ SEI is crucial for the protection of anode as it restricts transfer of electron from anode to electrolyte only allows the passage of Na^+^ ions to and from the anode. Thus, an ideal SEI should be electronic insulator and ionic conductor.[qv: 49b] Moreover, SEI should be stable so it also could prevent the dendritic growth of Na. There is no any focused study for SEI formation using different combination of organic solvents with different Na salt which can provide understanding about suitable anolyte. On the other hand, in most of studies, aqueous NaOH and KOH are usually used as catholyte. Due to the usage of aqueous electrolytes, the hybrid Na–air batteries have essential advantage over nonaqueous Na–O_2_ batteries thanks to the highly soluble discharge products (sodium hydroxide) which does not deposit into the pores of air electrodes, thus, enabling high performance hybrid Na–air battery. In addition to NaOH and KOH, Kang et al. modified the cell structure and used an acidic electrolyte instead of alkali electrolyte as catholyte.^44^ The acidic electrolyte also helped to improve the cell potential (3.94 V) and thus the specific energy (4418 Wh kg^−1^) as well. The charge–discharge reactions of acidic electrolyte‐based hybrid Na–air battery can be governed by following steps:

At anode

(7) $$\text{Na}\to {\text{Na}}^{+}+{\text{e}}^{-}\;\;E^{0}=+2.71\;\text{V}$$

At cathode

(8) $${\text{O}}_{2}+4{\text{H}}^{+}+4{\text{e}}^{-}\to 2{\text{H}}_{2}\text{O}\;\;E^{0}=+1.23\;\text{V}$$

Overall

(9) $$4\text{Na}+{\text{O}}_{2}+4{\text{H}}^{+}\to 4{\text{Na}}^{+}+2{\text{H}}_{2}\text{O}\;\;E^{0}=+3.94\;\text{V}$$

**Figure 8:**
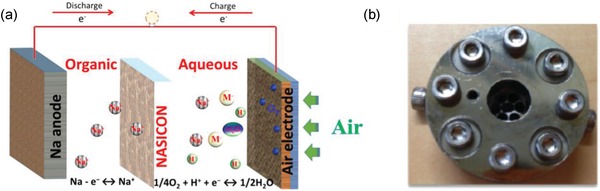
a) Scheme of a proposed acidic hybrid sodium−air cell. b) The digital image of assembled hybrid sodium−air battery. In acidic catholyte‐based hybrid cell, during discharge, oxygen gets reduced to form water. Reproduced with permission.^44^ Copyright 2018, American Chemical Society.

The assembled hybrid Na–air cell, as shown in **Figure** **8**, based on 0.1 m H_3_PO_4_ + 0.1 m Na_2_SO_4_ as catholyte exhibited maximum of 34.9 mW cm^−2^ power density and 896 mAh g^−1^ of discharge capacity with 0.3 V of overall overpotential gap. In acidic electrolyte systems, due to instability of NASICON at low pH, weak acids become an obvious choice but with the drawback of low ionic conductivity. Therefore, it always required to add a salt in weak acids to improve the conductivity of electrolyte.

### 3.4 Air Electrode

In rechargeable metal–air batteries, a perfect air electrode should have several essential features: i) a highly porous structure (material should be mesoporous, pore size varies from 2 to 50 nm) to promote the diffusion of air and electrolyte. But microporous materials with pore sizes up to 2 nm would block the diffusion of molecular oxygen; thus not able to access the catalytically active sites;^50^ ii) a high electrical conductivity; iii) a bifunctional activity, i.e., the capability to catalyze the charge (associated with OER) and discharge (associated with ORR) reactions, helps to improve energy efficiency, cyclic stability, and reduces the overpotentials. Nonetheless, the design and fabrication of bifunctional electrocatalyst constitute a challenging research area.^51^ In this view, major endeavors were dedicated into the synthesis and fabrication of appropriate air electrodes to lower the polarization of hybrid Na–air cell and thus enhance the energy density, electrical energy efficiency, and cyclic stability.

Hybrid Na–air battery was first demonstrated by Hayashi et al. using NASICON as a ceramic separator in a seminal study published in mid‐2013.[qv: 34b] Since, then there has been continuous growth in this area and various attempts have been made to improve the performance of hybrid Na–air battery by using different nanostructured air electrodes. Though Hayashi et al. did not study the rechargeability of hybrid Na–air cells, they systematically studied how to improve the cell performance and lower down the resistance.[qv: 34b] Typical hybrid Na–air cells contain different kinds of interfacial resistances at Na metal/organic electrolyte, organic electrolyte/ceramic separator, ceramic separator/aqueous electrolyte, and aqueous electrolyte/air cathode interfaces. The total cell resistance, which eventually lowers the power density, was reduced more than half (from 440 to 210 Ω) when appropriate concentration of electrolytes, optimized thickness of NASICON, and highly porous and conducting air electrode were used.[qv: 34b] Subsequently, authors achieved more than twice power density ≈10 mW cm^−2^ per effective area of ceramic separator with Pd‐catalyzed graphite plate as air cathode. Later, the same group reported a high energy density hybrid Na–air cell using Mn_3_O_4_ catalysts on porous carbon electrode as air cathode.^42^ Nevertheless, authors did not investigate the rechargeability of hybrid Na–air cell, but they achieved maximum of 21 mW cm^−2^ power density at room temperature which was the highest value ever reported for alkali metal–air cells. In late 2015, Hashimoto and Hayashi used nanoporous gold as air electrode and carried out a comparative study using aqueous and nonaqueous electrolytes.^52^ The authors used the same cell configuration except the catholyte and found that the cell with aqueous electrolyte is robust and displayed low overpotential gap (0.6 V for both charge and discharge), low resistance, and enormously improved power density (from 1.4 to 12.4 mW cm^−2^) and cyclic stability.

**Figure 9:**
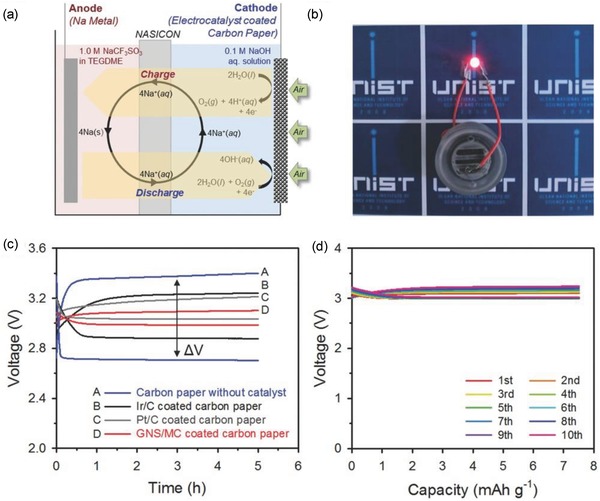
a) Schematic illustration of hybrid Na–air cell and redox processes involved in the cell. b) Image of hybrid Na–air cell using GNS/MC air electrode. c) Comparative galvanostatic charge–discharge profiles of hybrid Na–air cell using different air electrodes. d) Charge–discharge profile up to 10 cycles for hybrid air cell. Reproduced with permission.^37^ Copyright 2016, Wiley‐VCH.

At the same time, Sahgong et al. have demonstrated the rechargeability of hybrid Na–air cell using Pt/C as air electrode.^53^ Herein, the air electrode was designed by preparing the slurry through the mixing of catalyst Pt/C with binder polyvinylidene difluoride (PVDF) in *N*‐methyl‐2‐pyrrolidone (NMP) followed by its coating on a carbon paper. Using Pt/C as air electrode, the authors achieved 84.3% of energy efficiency and investigated the rechargeability of cell up to 18 cycles. However, the use of noble metal‐based air electrode is not promising due to their booming cost. Next, Cheon et al. synthesized graphitic nanoshell/mesoporous carbon nanohybrids (GNS/MC) as efficient bifunctional air electrode for hybrid Na–air battery.^37^ GNS/MC was synthesized by solid state nanocasting method using SBA‐15 silica as a template which showed exceptional catalytic activity and durability for ORR and OER in alkaline medium. The high electrocatalytic performance of the GNS/MC was attributed to the contributions of residual transition metal (Ni and Fe) entities, nitrogen‐doped defect‐rich graphitic nanoshells, and the high specific surface area of the mesoporous structure. After systematically investigating electrocatalysts, the authors fabricated a hybrid Na–air cell (scheme was presented in **Figure** **9**a; Figure 9b is the digital image of battery) which showed better overpotential gap of 115 mV than a cell made with Pt/C (179 mV) and Ir/C (364 mV) as air electrodes (Figure 9c). Furthermore, the authors also achieved a maximum of 78.2 mW g^−1^ in power density at 60 mA g^−1^ in current density. The rechargeability of hybrid cell using GNS/MC as air electrode was examined up to 10 cycles. The observed galvanostatic charge–discharge profile as presented in Figure 9d did not show any significant change in overpotential gap. Though GNS/MC showed lowest overpotential gap (0.115 V), yet the synthesis method is too complicated which cannot be employed for large‐scale production of the material.

**Figure 10:**
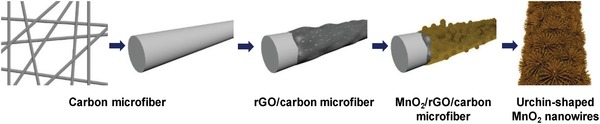
Schematic illustration of steps involved for growth of MnO_2_ NWs on rGO carbon paper. Reproduced with permission.^36^ Copyright 2016, Nature Publishing Group.

In the quest of cost‐effective air electrodes, Senthilkumar et al. explored the possibility to use cobalt phosphate as efficient bifunctional air electrode.^54^ The authors synthesized cobalt phosphate by simple precipitation followed by calcination method and functionalize a carbon paper electrode. The bifunctional electrocatalytic activity for ORR and OER was evaluated in alkali electrolyte by rotating ring‐disk electrode (RRDE) method. The electron transfer number associated with ORR was calculated to be ≈3.9 close to 4 which suggests that ORR occurred through a four‐electron transfer pathway, thus avoiding the formation of hydrogen peroxide. The electrocatalytic activity is associated with the formation of di μ‐oxo bridge within cobalt phosphate. The hybrid Na–air cell using cobalt phosphate as air electrode displays an overpotential gap of 0.23 V at 0.01 mA cm^−2^. The hybrid Na–air cell showed stability up 50 cycles at very low current density far from practical applications. Like in this last study, a typical design found in the literature for air electrodes include the coating of microporous carbon electrodes with a catalyst layer composed of electroactive (nano)particles bound together with a polymeric binder.^55^ Typically, polymeric binders, such as PVDF, are used to enhance the physical integrity of the coating and avoid delamination with the electrode substrate. There are several drawbacks of using polymeric binders: First, their electrical insulating character leads to the formation of a barrier for the transport of electrons between the carbon electrode and the electroactive particles; and for the transport of ions between the electrolyte and the electroactive particles. Second, some polymer binders such as PVDF was readily attacked by superoxide (O_2_^−^) formed as intermediate discharge product that leads the formation of fluorinated salt (NaF) and hydrogen peroxide hence limits the discharge capacity of the battery.^56^ Finally, the use of binder increases the total weight of the electrode and also requires an additional step to mix and combine the materials during the electrode preparation process.^55^ In that context, there is a motivation to find new strategies to avoid those polymeric binders. Khan et al. have grown α‐MnO_2_ nanowires on reduced graphene oxide (rGO) coated carbon paper and used this binder free electrode as air electrode for hybrid Na–air battery.^36^ This binder‐free electrode preparation involves coating of porous rGO on carbon microfiber followed by growth of α‐MnO_2_ nanowires on top of rGO coated carbon paper as shown in **Figure** **10**. Generally, MnO_2_ crystallizes in different phases, namely, α, β, and γ, and among them α‐MnO_2_ was found to have superior electrocatalytic activity than other phases.^57^ When MnO_2_ crystallizes in α‐phase, it tends to form 2 × 2 tunnel structures formed by edge‐sharing MnO_6_ and corner sharing MnO_6_ octahedra. These tunnel structures due to their size can accommodate positive ions to balance the negative charges during the electrocatalytic activity process. In this case, MnO_2_ nanowires crystallize in the α‐phase and display good electrocatalytic activity in alkaline solutions. The resulting hybrid Na–air cell displayed 0.7 V of overall overpotential gap at a current density of 15 mA g^−1^ when charged and discharged up to 25 h each. Interestingly, it was observed that urchin‐shaped α‐MnO_2_ on rGO coated carbon paper showed better performance than flake‐shaped MnO_2_ on rGO coated carbon paper. The urchin‐shaped MnO_2_ formed V‐shaped structures with highly exposed active sites which allow an enhanced diffusion and contact of the electrolyte to the air electrode, and an efficient transportation of Na^+^ ions and electrons. Hence, the morphology of the electroactive layer affects greatly the performance of the hybrid Na–air cell.

**Figure 11:**
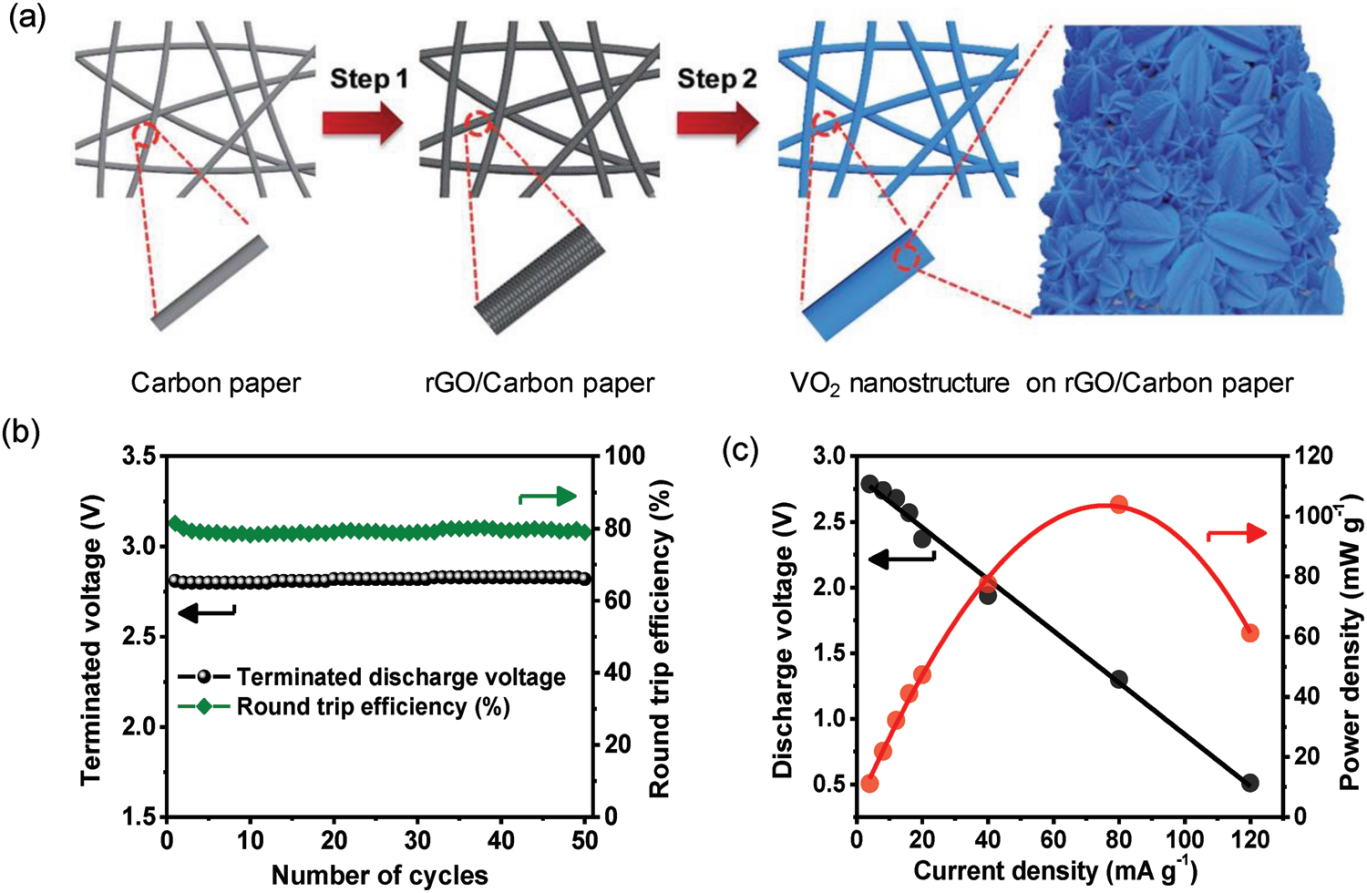
a) Schematic presentation of the steps involved during the synthesis of VO_2_ nanostructures on carbon paper. b) Cyclic stability in terms of terminated discharge potential and round trip efficiency. c) Power density plot of VO_2_ nanostructure used as air electrode in hybrid Na–air cell. Reproduced with permission.^43^ Copyright 2017, The Royal Society of Chemistry.

Later in 2016, in another work, Liang et al. exfoliated graphene from pyrolytic graphite and used this five layered exfoliated graphene as air electrode for hybrid Na–air cells.^58^ With that air electrode, the maximum power density (13.8 mW cm^−2^) was slightly higher than with a pyrolytic graphite air electrode (12.4 mW cm^−2^). Carambola‐shaped VO_2_ nanostructure was also utilized as air electrode for hybrid Na–air battery.^43^ The authors followed the same strategy as above, i.e., the carambola‐shaped VO_2_ were grown on rGO coated carbon paper, also resulting in a binder free air electrode (**Figure** **11**a). The hybrid Na–air cell displayed 0.64 V of overall overpotential gap, maximum of 104 mW g^−1^ power density, and good cyclic stability up to 50 cycles (Figure 11b,c). The good performance of the cell was attributed to the carambola‐shaped morphology of VO_2_ with well exposed arms facilitating a proper diffusion of air and electrolyte and preventing electrode polarization. Dual phase MnCo_2_O_4_ with N‐doped rGO was also explored as bifunctional electrocatalyst for hybrid battery.^59^ The composite material showed comparable ORR and higher OER performance than Pt/C which was credited to the dual‐phase of cobalt manganese spinel nanoparticles and N‐doping in rGO that improves the conductivity and catalytically active sites. The coupling of N‐doped rGO with MnCo_2_O_4_ helps to form a conducting network which improves the conductivity of the composite. Additionally, dopants in carbon matrix increase the active site which also enhances the catalytic activity of system.[qv: 2e] Thus, the fabricated cell displayed 2.75 V versus Na discharge, 3.14 V versus Na charge potential, and 0.39 V of overall overpotential gap with good cyclic stability up to 25 cycles.

**Figure 12:**
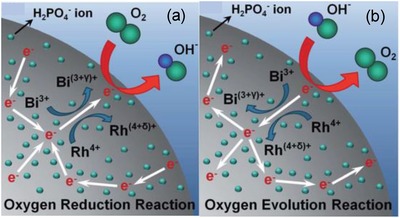
Schematic representation: a) ORR and b) OER catalytic origin of the P‐Bi_2_Rh_2_O_6.8_. Reproduced with permission.^62^ Copyright 2018, The Royal Society of Chemistry.

In another study, Khan et al. explored the possibility of using 3D SnS_2_ as air electrode for hybrid Na–air cell.^60^ On galvanostatic charge–discharge 3D SnS_2_ displayed 0.45 V discharge overpotential whereas, 0.07 V charge potential which leads to 0.52 V overall overpotential gap and 83% electrical energy efficiency. The vertically grown and self‐assembled ultrathin 3D SnS_2_ nanosheets of nanopetals displayed maximum of 300 mW g^−1^ power density and rechargeability up to 40 cycles. Various other materials such as Ni nanoparticles,^61^ bismuth rhodium oxide,^62^ Bi_2_Ru_2_O_7_,^63^ MOF‐derived carbon,^31^ Tl_2_Rh_2_O_7,_ 64 hollow double walled Mn_2_O_3_ nanocubes,^65^ and single crystalline Tl_2_Ru_2_O_7_ nanoparticles^66^ were also employed as air electrodes and studied systematically to improve the performance of hybrid Na–air cells (**Table** **4**). Kim et al. synthesized phosphate functionalized Bi_2_Rh_2_O_6.8_ as pyrochlore oxide and use it as a bifunctional air electrode for hybrid Na–air batteries.^62^ The synthesized material displayed the lowest potential gap (170 mV) between ORR and OER. This performance was attributed to the improvement in surface chemical activity due to the functionalization with phosphate ions. In functionalized P‐Bi_2_Rh_2_O_6.8_ due to lower Pauling electronegativity χ_r_ of P (χ_r_ = 2.19) compared to O (χ_r_ = 3.44), the —OPO(OH)_2_ surface groups promote a fast‐redox kinetics at the Bi and Rh centers during ORR and OER processes (**Figure** **12**). Authors proposed as “Therefore, oxidation of the Rh and Bi ions in P‐Bi_2_Rh_2_O_6.8_ was easily achieved because less energy is required to extract electrons during the ORR and OER, resulting in the fast catalyst kinetics and improved surface reactivity of the P‐Bi_2_Rh_2_O_6.8_.”^62^ The prepared material integrated in the air electrode leads to a cell with 0.17 V overpotential gap and 181.2 mW g^−1^ power density at 120 mA g^−1^ current density.

**Table 4:** Summary table associated with overpotential gap, power density, round trip efficiency, and cyclic stability for different air electrodes used in nonaqueous Na–O_2_ and hybrid Na–air cells

Air electrode	Overpotential^a)^ gap [V]	Power density	Round^b)^ trip efficiency [%]	Current density	Cycles	Refs.
Nonaqueous Na–O_2_ cell						
Diamond‐like carbon	≈1.5		62	0.1 C		67
Graphene nanosheet	1.6		61	300 mA g^−1^		[qv: 19l]
Carbon paste	1.8		56			68
Carbon‐fiber	0.2		92	120 µA cm^−2^	05	[qv: 6c]
Carbon nanotube paper	0.2		92	0.1 mA cm^−2^		[qv: 56c]
N‐doped graphene	1.4		61	75 mA g^−1^	03	[qv: 19i]
Carbon black	2.4		47	200 mA g^−1^	20	69
N‐CNT	1.95		53	75 mA g^−1^	50	[qv: 19k]
Pt@graphene	1.1		68	0.1 mA cm^−2^	10	70
CaMnO_3_/C	1.8		55	100 mA g^−1^	80	[qv: 19j]
CNT/Ni with NaI	1.5		55	0.05 mA cm^−2^	150	71
Mesoporous carbons	2.5		42	100 mA g^−1^	20	72
Ni_2_Co_2_O_4_ nanosheet/Ni foam	0.9			20 mA g^−1^	10	73
α‐MnO_2_ NWs	1.4			0.5 C	55	74
Co_3_O_4_ NWs/C/ferrocene	0.8		73	0.05 mA cm^−2^	570	75
CoO/CoP	0.7		76	500 mA g^−1^		22
CoB/CNT	0.7		76	100 mA g^−1^	74	76
m‐RuO_2_‐B‐rGO	0.7			0.05 mA cm^2^	100	77
Mesoporous boron‐doped onion‐like carbon			80	0.3 mA cm^−2^	125	78
N‐doped carbon						
Hybrid Na–air battery						
Pt mesh		4–5 mW cm^−2^				44
Pd‐catalyzed graphite plate		10 mW cm^−2^				44
Mn_3_O_4_ catalyzed carbon		21 mW cm^−2^				42
Nanoporous gold	≈0.7	12.4 mW cm^−2^	≈79	0.5 mA cm^−2^	22	52
Pt/C	0.53		84.3	0.025 mA cm^−2^	18	53
Co_3_(PO_4_)_2_	0.59		83	0.05 mA cm^−2^	50	54
Graphitic nanoshell/mesoporous carbon	0.115		96.2		10	37
MnO_2_ NWs/rGO/carbon paper	0.7		81	15 mA g^−1^	20	36
VO_2_/rGO/carbon paper	0.64	0.26 mW cm^−2^	81	4 mA g^−1^	50	43
MnCo_2_O_4_/N‐doped rGO	0.39		87	0.13 mA cm^−2^	20	59
3D SnS_2_ nanopetals	0.52	300 mW g^−1^	83	5 mA g^−1^	40	60
Mn_3_O_4_/C	0.7	27.6 mW cm^−2^	≈78	1 mA cm^−2^	5	79
Exfoliated graphene sheet		13.8 mW cm^−2^				58
MOF derived NCNTs	0.3		87	0.1 mA cm^−2^	35	31
NCNTs	0.77		77	0.1 mA cm^−2^		
Co‐CNTs	0.67		80	0.1 mA cm^−2^		
RuO_2_	0.5		84	0.1 mA cm^−2^		
MWCNTs	0.9		74	0.1 mA cm^−2^		
Bi_2_Ru_2_O_7_	0.211	156.3 mW g^−1^	93.58	0.01 mA cm^−2^	50	63
Tl_2_Rh_2_O_7_	0.208	159.9 mW g^−1^	93.65	0.01 mA cm^−2^	50	64
Phosphate‐ion functionalized Bi_2_Rh_2_O_6.8_	0.17	181.2 mW g^−1^	94.9	0.01 mA cm^−2^	50	62
Pt/C^c)^	0.3	34.9 mW cm^−2^	90	0.13 mA cm^−2^	30	44
40 wt% Pt/C^d)^	0.14	39 mW cm^−2^	95.3	0.1 mA cm^−2^	80	47
Ni nanoparticle	0.57		80.3	0.1 mA cm^−2^	100	61
Hollow double walled Mn_2_O_3_ nanocubes	0.33	200 mW g^−1^	90	5 mA g^−1^	75	65
N,S‐doped carbon nanospheres^e)^	0.56	203 mW g^−1^	84	5 mA g^−1^	100	[qv: 2e]

^a)^ Overpotential gap calculation based on charge–discharge voltage plateau

^b)^ Round trip efficiency calculation based on charge–discharge voltage plateau

^c)^ Acidic electrolyte‐based hybrid Na–air cell

^d)^ Anode is liquid anode (metallic Na is dissolved in biphenyl)

^e)^ The catholyte here was seawater and air were continuously purged in catholyte.

All in all, there has been significant research effort to find suitable air electrode for hybrid Na–air cells. Despite clear improvements of the performance of the batteries, they still lack cyclic stability to become commercially viable. Moreover, the power density is limited, and this is mainly due to the poor OER electrocatalytic activity at the air electrode.

## 4 Conclusion and Future Prospect

As described in this progress report, there is a strong competition between the two key sodium battery technologies: nonaqueous Na–O_2_ and hybrid Na–air batteries. Nonaqueous Na–O_2_ batteries are safer than hybrid Na–air due to the usage of organic electrolyte as anolyte and catholyte. Indeed, the reaction of water with metal sodium can be explosive. However, the use of an organic electrolyte leads to a nonconductive and insoluble discharge product that deposits in the pores of the air electrode and progressively covers the active reaction sites and blocks the O_2_ diffusion pathway. This detrimental process increases the overpotentials specifically during charging, which is accompanied by a reduction of the power density and worsens the cyclic stability. In addition, organic electrolytes are more expensive, flammable, and with a certain level of toxicity. Most of nonaqueous Na–O_2_ cell required usage of either pure and dry oxygen or oxygen diffusion membrane which will also increase the cost of the cell. However, the research in the area of hybrid Na–air battery is gaining momentum since its first reports in 2013. The hybrid Na–air cell uses less organic electrolyte and consumes air as oxygen source thereby immediately reducing the cost, in contrast to aprotic Na–O_2_ cell. In mixed electrolyte system (aqueous electrolyte and aprotic electrolyte), the formed discharge product is soluble in water thus diminishing the clogging and electronic conductivity issues related to organic electrolytes. Unlike nonaqueous Na–O_2_ battery, hybrid Na–air battery offers several advantages such as low overpotential, high energy efficiency, low electrode polarization, good cyclic stability, and use of ambient air as source of oxygen. Though hybrid Na–air batteries offer potential solution to nonaqueous Na–O_2_, yet some challenges remain which needs to be investigated and optimized for future practical applications. NASICON, the solid electrolyte membrane, is an essential component in hybrid Na–air battery. However, the interfacial chemistry between the organic electrolyte and NASICON is not known yet but need to be understood and controlled because it typically affects the resistance of the cell. Additionally, the use of NASICON has limitations for charge–discharge processes of the cells at high current densities (for practical application current density should be 100 mA cm^−2^) and its ionic conductivity requires further improvement. In most of the studies associated with hybrid Na–air cells, metallic Na has been used as anode and it is well known that metallic sodium tends to form dendritic structure upon deposition and stripping process. One challenge would be to design metallic anodes with artificial SEI or to modify its surface properties to prevent the formation of dendrites. The hybrid system requires two different electrolytes. At the present time, NaOH and KOH appear to be good choices for catholyte, but the proper selections of an organic electrolyte and respective Na salt need to be addressed. The organic electrolyte decides the formation of stable SEI on anode which will also help to prevent the growth of dendrites and eventually improves the performance of the cell. Moreover, in the existing hybrid Na–air battery, the evaporation of water is an issue leading to drying the solid electrolyte membrane and deactivating the air electrode. The consequence is an enhancement of the resistance and a degradation of the catalytic processes. Hence water evaporation must be prevented with proper encapsulation. Last, the proper selection and design of the air electrode seems to be a key topic to improve the performance of hybrid Na–air cell. The air electrode should catalyze both OER and ORR associated with charge and discharge of the cell, hence key electrocatalyst functionalizing the air electrode should be bifunctional in nature. In addition to its bifunctional character, the catalyst should be highly porous and interconnected for efficient diffusion of electrolyte ions and oxygen in order to have a large triple point area (gas, solid, liquid) where electrocatalytic reactions take place. Various metal oxides or metal oxide in combination with carbon nanostructures, or carbon materials alone, have been studied for hybrid Na–air cells. Today, most air electrodes suffer from high charge potential possibly due to the poor catalytic activity for OER. Therefore, in the future it is highly desirable to design air electrode which can efficiently catalyze OER and ORR.

In summary, hybrid Na–air batteries have emerged as new types of energy storage devices with promising energy and power density. They showed fundamental advantages over nonaqueous Na–O_2_ battery as well as better technical specifications. However, for practical and large‐scale applications (such as electric vehicles, hybrid electric vehicles, and grid backups), more understanding and designs are required not only in the area of air‐electrodes but also separators, anodes, and electrolytes. The hybrid Na–air battery technology is still in its nascent stage, but we believe that the collaborative work performed in this research community will lead to a new commercial battery technology.

## Conflict of Interest

The authors declare no conflict of interest.
